# Crystal structures of two (±)-*exo*-*N*-isobornyl­acetamides

**DOI:** 10.1107/S2056989015015984

**Published:** 2015-09-12

**Authors:** Dmitrijs Stepanovs, Daniels Posevins, Maris Turks

**Affiliations:** a Latvian Institute of Organic Synthesis, Aizkraukles 21, Riga, LV-1006, Latvia; b Institute of Technology of Organic Chemistry, Faculty of Materials Science and Applied Chemistry, Riga Technical University, P. Valdena 3/7, Riga, LV-1048, Latvia

**Keywords:** crystal structure, Ritter reaction, (±)-*exo*-*N*-isobornylacetamides, polymorph, hydrogen bonding

## Abstract

The title compounds consist of a bornane skeleton with attached acetamide, C_12_H_21_NO (±)-(**1**) {systematic name: (±)-*N*-[(1*RS*,2*RS*,4*RS*)-1,7,7-tri­methylbi­cyclo­[2.2.1]heptan-2-yl]acetamide}, and chloro­acetamide, C_12_H_20_ClNO (±)-(**2**) {systematic name: (±)-2-chloro-*N*-[(1*RS*,2*RS*,4*RS*)-1,7,7-tri­methylbi­cyclo­[2.2.1]heptan-2-yl]­acetamide}, functionalities to the 2-*exo*-position. The crystal structure of the first monoclinic polymorph of (±)-(**1**) has been reported previously [Ung *et al.* (2014 ▸). *Monatsh. Chem.* **145**, 983–992]. Compound (±)-(**1**) crystallizes in the space group *P*2_1_/*n* with two independent mol­ecules in the asymmetric unit, in contrast to the above-mentioned polymorph which crystallized in the space group *C*2/*c* with one mol­ecule in the asymmetric unit. In the title compounds, the bicyclic bornane moieties have normal geometries. In the crystals of both compounds, mol­ecules are linked by N—H⋯O hydrogen bonds, reinforced by C—H⋯O contacts, forming *trans*-amide chains propagating along the *a*-axis direction. In the case of compound (±)-(**1**), neighbouring chains are linked by further C—H⋯O contacts, forming double-chain ribbons along [100].

## Abstract 2

The title compounds consist of a 1,7,7-tri­methylbi­cyclo­[2.2.1]heptane (bornane or camphane) skeleton which is decorated with acetamide for (±)-(**1**) and chloro­acetamide for (±)-(**2**), functionalities. In the crystals of both compounds, mol­ecules are linked *via* N—H⋯O hydrogen bonds, reinforced by C—H⋯O contacts, forming chains propagating along the *a* axis.

## Chemical context   

Isobornyl­amine-derived amides have recently been described as useful anti­mycobacterial agents (Stavrakov *et al.*, 2014*a* ▸,*b* ▸). Promising biological activity profiles have been also discovered for other bornane derivatives such as 2-aryl­bornanes (Duclos *et al.*, 2008 ▸), camphor oximes (Schenone *et al.*, 2000 ▸), bornyl (3,4,5-trihy­droxy)-cinnamate (Steinbrecher *et al.*, 2008 ▸) and others. There is no doubt that isobornyl­amine derivatives are chemically related to terpenoids camphor (Seebaluck *et al.*, 2015 ▸) and borneol (Horváthová *et al.*, 2012 ▸), which are well known for their biological activities. On the other hand, compounds containing the bornane skeleton are frequently used as chiral building blocks for various ligands, catalysts and chiral auxiliaries (Chelucci, 2006 ▸; Langlois & Kouklovsky, 2009 ▸; Ramón & Yus, 2007 ▸). In light of the aforementioned facts, there is a vast inter­est in developing new synthetic protocols for the synthesis of compounds of this class and in their structural studies. We have recently reported an application of the Ritter reaction (Jiang *et al.*, 2014 ▸) in the synthesis of amide-derivatized heterocycles (Turks *et al.*, 2012 ▸). Hence, we identified the possibility to obtain isobornyl­amine derived amides (±)-(**1**) and (±)-(**2**) from borneol in the direct Ritter reaction. When the optically active (−)-borneol was submitted to standard Ritter reaction conditions, the expected compounds were isolated in acceptable yields albeit in the racemic form. A similar type of racemization due to a 6,2-hydride shift was described in the Ritter reaction of (−)-bornyl acetate (Hanzawa *et al.*, 2012 ▸.). Previously, compounds (±)-(**1**) and (±)-(**2**) have been obtained as side products in a cationic rearrangement of (−)-β-pinene in the presence of the corres­ponding nitriles (Ung *et al.*, 2014 ▸).
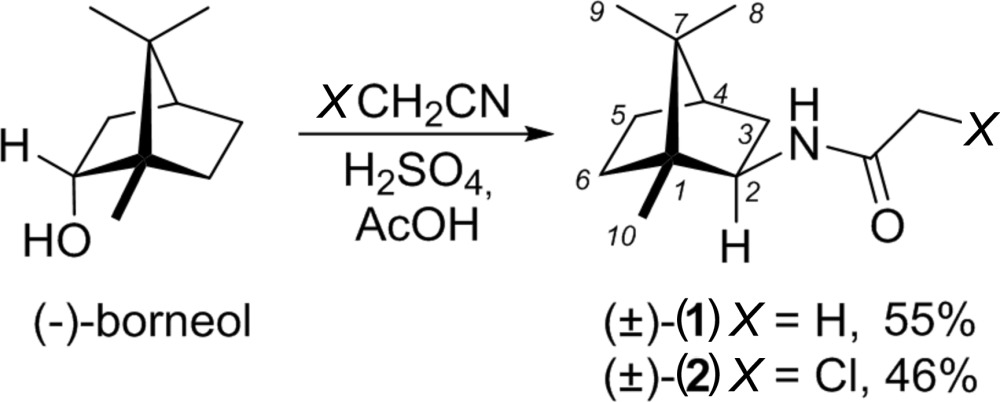


## Structural commentary   

**Figure 1:**
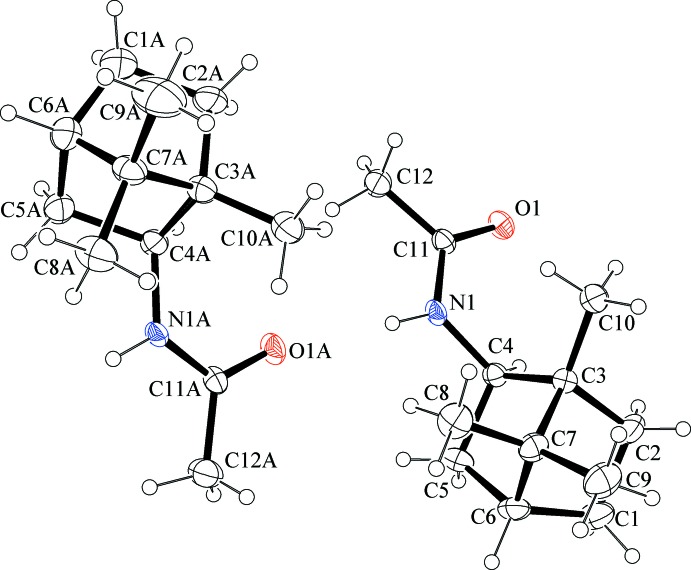
The mol­ecular structure of the two independent mol­ecules of compound (±)-(**1**), showing the atom labelling. Displacement ellipsoids are drawn at the 50% probability level.

**Figure 2:**
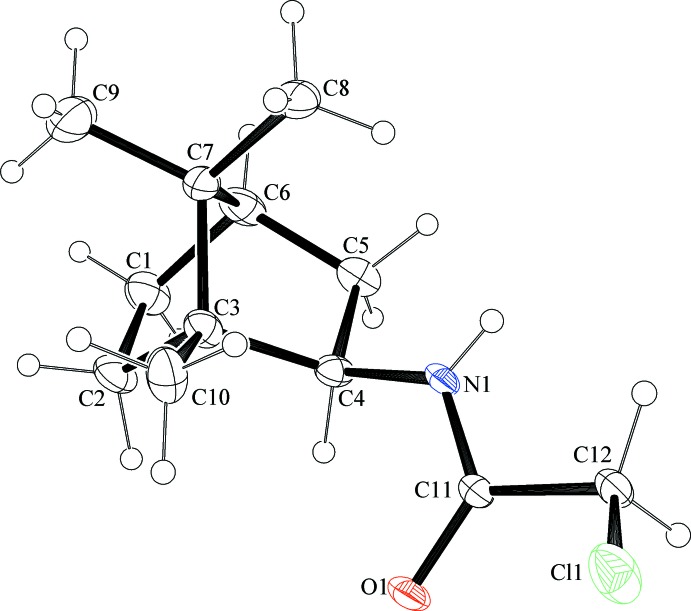
The mol­ecular structure of compound (±)-(**2**), showing the atom labelling. Displacement ellipsoids are drawn at the 50% probability level.

The title compounds consist of a 1,7,7-tri­methylbi­cyclo[2.2.1]heptane (bornane or camphane) skeleton with attached acetamide [(±)-(**1**)] and chloro­acetamide [(±)-(**2**)] functionalities in the 2-*exo*-position. The asymmetric unit of compound (±)-(**1**) (Fig. 1 ▸) contains two independent mol­ecules having coincident geometry (r.m.s. deviation 0.057 Å). Compound (±)-(**2**) (Fig. 2 ▸) contains one mol­ecule in the asymmetric unit. The bond lengths and angles in both compounds are close to those observed for the first monoclinic polymorph of compound (±)-(**1**) (Ung *et al.*, 2014 ▸).

## Supra­molecular features   

**Figure 3:**
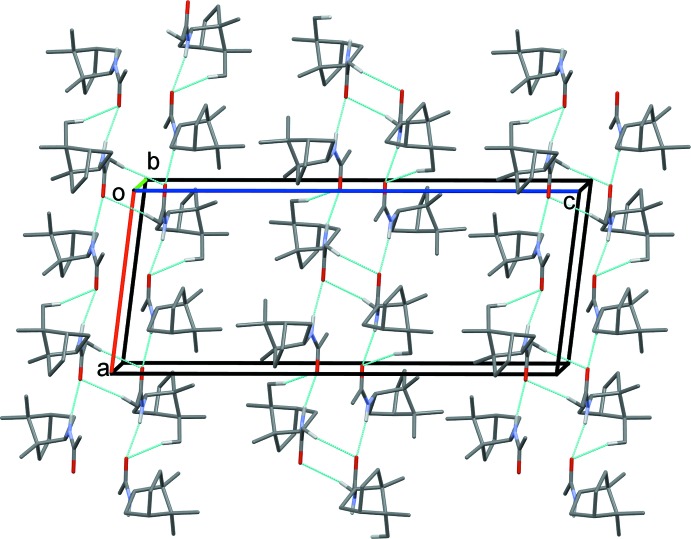
The crystal packing of compound (±)-(**1**), viewed along the *b* axis. Hydrogen bonds are shown as dashed lines (see Table 1 ▸ for details). For clarity, only H atoms involved in these inter­actions have been included.

In the crystals of both compounds, mol­ecules are linked by N—H⋯O hydrogen bonds, reinforced by C—H⋯O contacts, forming *trans*-amide chains propagating along the *a* axis direction (Figs. 3 ▸ and 4 ▸ and Tables 1 ▸ and 2 ▸). In the case of compound (±)-(**1**), neighbouring chains are linked by further C—H⋯O contacts, forming ribbons along [100]; see Fig. 3 ▸ and Table 1 ▸.

## Database survey   

A search of the Cambridge Structural Database (Version 5.36; Groom & Allen, 2014 ▸) for substituted bornanes gave 1517 hits (excluding organometallics). 119 structures are substituted at the 2-position. Only two of these are amides, *viz*. the previously reported polymorph of (±)-(**1**) (LOPQEO: Ung *et al.*, 2014 ▸) and 2,2,2-triphenyl-*N*-(1,7,7-tri­methylbi­cyclo­[2.2.1]hept-2-yl)acetamide (TOQWED: Prusinowska *et al.*, 2015 ▸).

## Synthesis and crystallization   

**Compound (**±**)-(1):** (−)-Borneol (463 mg, 3 mmol, 1 equiv.) was added to a stirred solution of aceto­nitrile (790 µL, 15 mmol, 5.0 equiv.) in glacial acetic acid (7.0 ml) and conc. H_2_SO_4_ (3.07 g, 30 mmol, 10.0 equiv.). The resulting reaction mixture was stirred at 343 K for 16 h (TLC control). The reaction mixture was cooled to 273 K and poured into a vigorously stirred 10% aqueous solution of NaOH (30–40 mL) at 273 K. Ethyl acetate (30 mL) was added and the phases were separated. The aqueous phase was extracted with ethyl acetate (3 × 20 mL). The combined organic phase was washed with brine, dried over anhydrous Na_2_SO_4_, filtered and evaporated under reduced pressure. The resulting residue was purified by silica gel column chromatography to provide (±)-(**1**) (yield: 319 mg, 55%). The NMR data of (±)-(**1**) corres­pond fully to those reported earlier (Ung *et al.*, 2014 ▸): ^1^H NMR (300 MHz, CDCl_3_) δ (p.p.m.): 5.44 (*br s*, 1H), 3.87 (*td*, *J* = 9.0, 5.2 Hz, 1H), 1.97–1.77 (*m*, 4H), 1.73–1.61 (*m*, 2H), 1.60–1.46 (*m*, 2H), 1.32–1.20 (*m*, 1H), 1.18–1.07 (*m*, 1H), 0.88 (*s*, 3H), 0.81 (*s*, 3H), 0.80 (*s*, 3H); ^13^C NMR (75.5 MHz, CDCl_3_) δ (p.p.m.): 169.35, 56.81, 48.87, 47.14, 44.92, 39.15, 36.02, 27.06, 23.69, 20.38, 20.35, 11.77. GC–MS (C_12_H_21_NO): *t_R_* = 5.92 min; *m*/*z*: calculated 195.2; found 195.1. (GC–MS method: column: HP5 (5% phenyl methyl siloxane), 30 m × 0.25 mm ID, 0.25 µm; column temp.: 323 K (hold for 2 min) to 583 K at 323 K min^−1^ (hold at 583 K for 3 min); injector/detector: 523 K/503 K; carrier gas: helium at 1.0 mL min^−1^, linear velocity; injection mode: splitless (solvent delay: 3 min); injection volume: 1 µL). X-ray quality single crystals were obtained by slow evaporation of a solution of (±)-(**1**) in hexa­nes/ethyl acetate (2:1).

**Compound (**±**)-(2)**: (−)-Borneol (463 mg, 3 mmol, 1 equiv.) was added to a stirred solution of chloro­aceto­nitrile (950 µL, 15 mmol, 5.0 equiv.) in glacial acetic acid (7.0 ml) and conc. H_2_SO_4_ (3.07 g, 30 mmol, 10.0 equiv.). The resulting reaction mixture was stirred at 343 K for 16 h (TLC control). The reaction mixture was cooled to 273 K and poured into a vigorously stirred 10% aqueous solution of NaOH (30-40 mL) at 273 K. Ethyl acetate (30 mL) was added and the phases were separated. The aqueous phase was extracted with ethyl acetate (3 × 20 mL). The combined organic phase was washed with brine, dried over anhydrous Na_2_SO_4_, filtered and evaporated under reduced pressure. The resulting residue was purified by silica gel column chromatography to provide (±)-(**2**) (yield: 318 mg, 46%). The NMR data of (±)-(**2**) fully correspond to those reported earlier (Ung *et al.*, 2014 ▸): ^1^H NMR (300 MHz, CDCl_3_) δ (p.p.m.): 6.63 (*br s*, 1H), 4.03 (*d*, *J* = 1.5 Hz, 2H), 3.88 (*td*, *J* = 9.1, 4.9 Hz, 1H), 1.87 (*dd*, *J* = 13.3, 9.1 Hz, 1H), 1.80–1.52 (*m*, 4H), 1.35–1.23 (*m*, 1H), 1.22–1.10 (*m*, 1H), 0.94 (*s*, 3H), 0.86–0.83 (*m*, 6H); ^13^C NMR (75.5 MHz, CDCl_3_) δ (p.p.m.): 164.97, 57.12, 48.69, 47.21, 45.01, 43.00, 39.01, 35.95, 27.10, 20.33, 20.19, 11.82. GC–MS (C_12_H_20_^35^ClNO): *t_R_* = 6.21 min; *m*/*z*: calculated 229.1; found 229.1. (GC–MS method: *vide supra*). X-ray quality single crystals were obtained by slow evaporation of a solution of (±)-(**2**) in hexa­nes/ethyl acetate (2:1).

## Refinement   

Crystal data, data collection and structure refinement details are summarized in Table 3 ▸. For both compounds, the H atom on the amino group were located in difference Fourier maps and freely refined, and the C-bound H atoms were positioned geometrically and refined as riding on their parent atoms: C—H = 0.93–0.97 Å with *U*_iso_(H) = 1.5*U*_eq_(C) for methyl H atoms and 1.2*U*_eq_(C) for other H atoms. Reflection (0,1,1) whose intensity was affected by the beam-stop was removed from the final refinement of compound (±)-(**1**).

## Supplementary Material

Crystal structure: contains datablock(s) 1, 2, Global. DOI: 10.1107/S2056989015015984/su5191sup1.cif

Structure factors: contains datablock(s) 1. DOI: 10.1107/S2056989015015984/su51911sup2.hkl

Supporting information file. DOI: 10.1107/S2056989015015984/su51911sup4.cml

Structure factors: contains datablock(s) 2. DOI: 10.1107/S2056989015015984/su51912sup3.hkl

Supporting information file. DOI: 10.1107/S2056989015015984/su51912sup5.cml

CCDC references: 1420761, 1420760

Additional supporting information:  crystallographic information; 3D view; checkCIF report

## Figures and Tables

**Figure 4:**
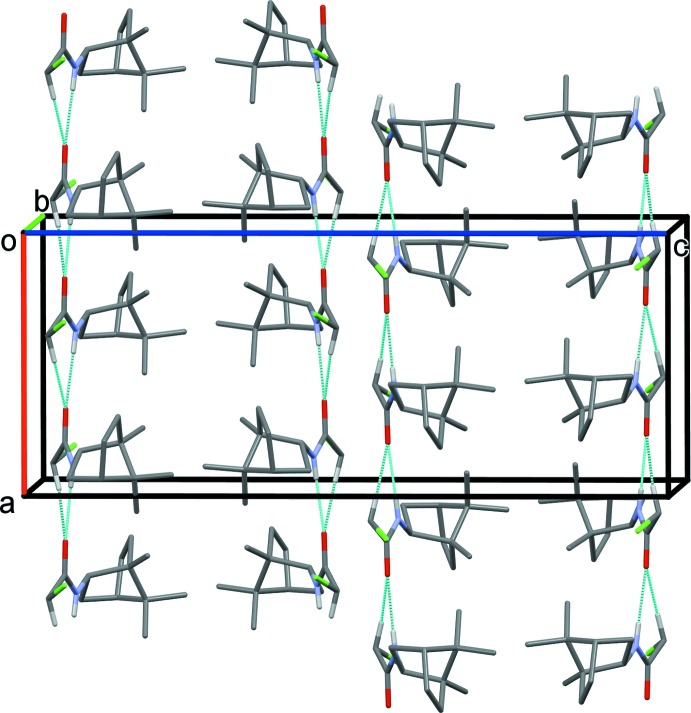
The crystal packing of compound (±)-(**2**), viewed along the *b* axis. Hydrogen bonds are shown as dashed lines (see Table 2 ▸ for details). For clarity, only H atoms involved in these inter­actions have been included.

**Table 1:** Hydrogen-bond geometry (Å, °) for (±)-(**1**)

*D*—H⋯*A*	*D*—H	H⋯*A*	*D*⋯*A*	*D*—H⋯*A*
N1—H1*N*⋯O1*A* ^i^	0.85 (2)	2.06 (2)	2.900 (2)	170 (2)
N1*A*—H1*AN*⋯O1^ii^	0.87 (2)	2.03 (2)	2.886 (2)	172 (2)
C8*A*—H8*A*1⋯O1^ii^	0.98	2.57	3.524 (3)	165
C12—H12*C*⋯O1^ii^	0.98	2.52	3.468 (3)	164

Symmetry codes: (i) 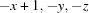; (ii) 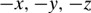.

**Table 2:** Hydrogen-bond geometry (Å, °) for (±)-(**2**)

*D*—H⋯*A*	*D*—H	H⋯*A*	*D*⋯*A*	*D*—H⋯*A*
N1—H1⋯O1^i^	0.79 (3)	2.21 (3)	2.983 (2)	168 (2)
C12—H12*A*⋯O1^i^	0.97	2.36	3.238 (3)	151

Symmetry code: (i) 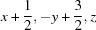.

**Table 3:** Experimental details

	(±)-(**1**)	(±)-(**2**)
Crystal data		
Chemical formula	C_12_H_21_NO	C_12_H_20_ClNO
*M*_r_	195.30	229.74
Crystal system, space group	Monoclinic, *P*2_1_/*n*	Orthorhombic, *P* *c* *a* *b*
Temperature (K)	173	173
*a*, *b*, *c* (Å)	9.6820 (6), 10.6540 (3), 23.3676 (7)	9.6852 (2), 10.7589 (3), 23.7261 (8)
α, β, γ (°)	90, 97.184 (10), 90	90, 90, 90
*V* (Å^3^)	2391.49 (19)	2472.31 (12)
*Z*	8	8
Radiation type	Mo *K*α	Mo *K*α
μ (mm^−1^)	0.07	0.29
Crystal size (mm)	0.18 × 0.12 × 0.09	0.35 × 0.10 × 0.09

Data collection		
Diffractometer	Nonius KappaCCD	Nonius KappaCCD
No. of measured, independent and observed [*I* > 2σ(*I*)] reflections	7908, 4320, 2637	6757, 3611, 1854
*R*_int_	0.056	0.097
(sin θ/λ)_max_ (Å^−1^)	0.600	0.704

Refinement		
*R*[*F* ^2^ > 2σ(*F* ^2^)], *wR*(*F* ^2^), *S*	0.065, 0.159, 1.05	0.073, 0.160, 1.02
No. of reflections	4320	3611
No. of parameters	269	143
H-atom treatment	H atoms treated by a mixture of independent and constrained refinement	H atoms treated by a mixture of independent and constrained refinement
Δρ_max_, Δρ_min_ (e Å^−3^)	0.37, −0.20	0.51, −0.38

Computer programs: *KappaCCD Server Software* (Nonius, 1997 ▸), *DENZO* and *SCALEPACK* (Otwinowski & Minor, 1997 ▸), *SIR2011* (Burla *et al.*, 2012 ▸), *ORTEP-3 for Windows* (Farrugia, 2012 ▸), *Mercury* (Macrae *et al.*, 2008 ▸), *SHELXL97* (Sheldrick, 2008 ▸), *PLATON* (Spek, 2009 ▸) and *publCIF* (Westrip, 2010 ▸).

## supplementary crystallographic information

### (1) (±)-N-[(1RS,2RS,4RS)-1,7,7-Trimethylbicyclo[2.2.1]heptan-2-yl]acetamide Crystal data

**Table:**

C_12_H_21_NO	*F*(000) = 864
*M**_r_* = 195.30	*D*_x_ = 1.085 Mg m^−^^3^
Monoclinic, *P*2_1_/*n*	Mo *K*α radiation, λ = 0.71073 Å
Hall symbol: -P 2yn	Cell parameters from 23028 reflections
*a* = 9.6820 (6) Å	θ = 1.0–30.0°
*b* = 10.6540 (3) Å	µ = 0.07 mm^−^^1^
*c* = 23.3676 (7) Å	*T* = 173 K
β = 97.184 (10)°	Plate, colourless
*V* = 2391.49 (19) Å^3^	0.18 × 0.12 × 0.09 mm
*Z* = 8	

### (1) (±)-N-[(1RS,2RS,4RS)-1,7,7-Trimethylbicyclo[2.2.1]heptan-2-yl]acetamide Data collection

**Table:**

Nonius KappaCCD diffractometer	2637 reflections with *I* > 2σ(*I*)
Radiation source: fine-focus sealed tube	*R*_int_ = 0.056
Graphite monochromator	θ_max_ = 25.3°, θ_min_ = 2.2°
φ and ω scan	*h* = −11→11
7908 measured reflections	*k* = −12→11
4320 independent reflections	*l* = −28→27

### (1) (±)-N-[(1RS,2RS,4RS)-1,7,7-Trimethylbicyclo[2.2.1]heptan-2-yl]acetamide Refinement

**Table:**

Refinement on *F*^2^	Primary atom site location: structure-invariant direct methods
Least-squares matrix: full	Secondary atom site location: difference Fourier map
*R*[*F*^2^ > 2σ(*F*^2^)] = 0.065	Hydrogen site location: inferred from neighbouring sites
*wR*(*F*^2^) = 0.159	H atoms treated by a mixture of independent and constrained refinement
*S* = 1.05	*w* = 1/[σ^2^(*F*_o_^2^) + (0.0684*P*)^2^ + 0.5097*P*] where *P* = (*F*_o_^2^ + 2*F*_c_^2^)/3
4320 reflections	(Δ/σ)_max_ < 0.001
269 parameters	Δρ_max_ = 0.37 e Å^−^^3^
0 restraints	Δρ_min_ = −0.20 e Å^−^^3^

### (1) (±)-N-[(1RS,2RS,4RS)-1,7,7-Trimethylbicyclo[2.2.1]heptan-2-yl]acetamide Special details

**Table:**

Geometry. All esds (except the esd in the dihedral angle between two l.s. planes) are estimated using the full covariance matrix. The cell esds are taken into account individually in the estimation of esds in distances, angles and torsion angles; correlations between esds in cell parameters are only used when they are defined by crystal symmetry. An approximate (isotropic) treatment of cell esds is used for estimating esds involving l.s. planes.
Refinement. Refinement of F^2^ against ALL reflections. The weighted R-factor wR and goodness of fit S are based on F^2^, conventional R-factors R are based on F, with F set to zero for negative F^2^. The threshold expression of F^2^ > 2sigma(F^2^) is used only for calculating R-factors(gt) etc. and is not relevant to the choice of reflections for refinement. R-factors based on F^2^ are statistically about twice as large as those based on F, and R- factors based on ALL data will be even larger.

### (1) (±)-N-[(1RS,2RS,4RS)-1,7,7-Trimethylbicyclo[2.2.1]heptan-2-yl]acetamide Fractional atomic coordinates and isotropic or equivalent isotropic displacement parameters (Å^2^)

**Table:**

	*x*	*y*	*z*	*U*_iso_*/*U*_eq_	
O1	−0.02258 (14)	0.12729 (16)	0.06523 (7)	0.0422 (5)	
N1	0.18985 (18)	0.21667 (19)	0.07437 (8)	0.0296 (5)	
H1N	0.276 (2)	0.204 (2)	0.0728 (9)	0.036 (7)*	
C1	0.1084 (3)	0.6083 (3)	0.09760 (13)	0.0543 (8)	
H1A	0.0660	0.6280	0.0579	0.065*	
H1B	0.1171	0.6865	0.1206	0.065*	
C2	0.0227 (2)	0.5082 (2)	0.12569 (12)	0.0474 (7)	
H2A	−0.0068	0.5399	0.1621	0.057*	
H2B	−0.0610	0.4837	0.0993	0.057*	
C3	0.1236 (2)	0.3969 (2)	0.13719 (10)	0.0346 (6)	
C4	0.1376 (2)	0.3440 (2)	0.07640 (10)	0.0311 (6)	
H4	0.0440	0.3475	0.0529	0.037*	
C5	0.2338 (2)	0.4415 (2)	0.05134 (11)	0.0402 (6)	
H5A	0.3248	0.4039	0.0460	0.048*	
H5B	0.1898	0.4752	0.0140	0.048*	
C6	0.2501 (2)	0.5434 (2)	0.09772 (11)	0.0436 (7)	
H6	0.3292	0.6024	0.0947	0.052*	
C7	0.2638 (2)	0.4669 (2)	0.15414 (10)	0.0385 (6)	
C8	0.3926 (2)	0.3821 (3)	0.16342 (12)	0.0478 (7)	
H8A	0.3995	0.3333	0.1283	0.072*	
H8B	0.3844	0.3248	0.1956	0.072*	
H8C	0.4762	0.4339	0.1722	0.072*	
C9	0.2675 (3)	0.5485 (3)	0.20856 (12)	0.0583 (8)	
H9A	0.3534	0.5981	0.2135	0.087*	
H9B	0.2645	0.4945	0.2423	0.087*	
H9C	0.1870	0.6050	0.2046	0.087*	
C10	0.0805 (3)	0.3019 (3)	0.17942 (12)	0.0481 (7)	
H10D	0.0789	0.3419	0.2171	0.072*	
H10E	0.1471	0.2323	0.1831	0.072*	
H10F	−0.0126	0.2700	0.1654	0.072*	
C11	0.1054 (2)	0.1169 (2)	0.06847 (9)	0.0318 (6)	
C12	0.1737 (2)	−0.0091 (2)	0.06613 (12)	0.0422 (7)	
H12A	0.1673	−0.0544	0.1022	0.063*	
H12B	0.2719	0.0022	0.0610	0.063*	
H12C	0.1267	−0.0574	0.0337	0.063*	
O1A	0.52606 (14)	−0.16700 (16)	−0.05366 (7)	0.0413 (5)	
N1A	0.31245 (18)	−0.08247 (18)	−0.07554 (8)	0.0297 (5)	
H1AN	0.223 (2)	−0.092 (2)	−0.0758 (9)	0.035 (6)*	
C1A	0.3751 (3)	0.2963 (3)	−0.12778 (15)	0.0615 (8)	
H1A1	0.4035	0.3332	−0.0892	0.074*	
H1A2	0.3675	0.3640	−0.1570	0.074*	
C2A	0.4775 (2)	0.1949 (2)	−0.14177 (13)	0.0523 (8)	
H2A1	0.5552	0.1864	−0.1102	0.063*	
H2A2	0.5161	0.2145	−0.1780	0.063*	
C3A	0.3881 (2)	0.0737 (2)	−0.14821 (11)	0.0396 (6)	
C4A	0.3603 (2)	0.0434 (2)	−0.08641 (10)	0.0343 (6)	
H4A	0.4474	0.0600	−0.0597	0.041*	
C5A	0.2472 (3)	0.1450 (3)	−0.07462 (12)	0.0463 (7)	
H5A1	0.1567	0.1053	−0.0702	0.056*	
H5A2	0.2786	0.1956	−0.0399	0.056*	
C6A	0.2383 (3)	0.2240 (3)	−0.12928 (14)	0.0568 (8)	
H6A	0.1537	0.2786	−0.1354	0.068*	
C7A	0.2459 (2)	0.1260 (3)	−0.17734 (11)	0.0418 (7)	
C8A	0.1271 (2)	0.0305 (3)	−0.18521 (12)	0.0530 (8)	
H8A1	0.1113	−0.0029	−0.1475	0.079*	
H8A2	0.1518	−0.0383	−0.2098	0.079*	
H8A3	0.0421	0.0715	−0.2033	0.079*	
C9A	0.2545 (3)	0.1849 (4)	−0.23710 (14)	0.0819 (11)	
H9A1	0.1685	0.2313	−0.2495	0.123*	
H9A2	0.2664	0.1184	−0.2650	0.123*	
H9A3	0.3340	0.2424	−0.2347	0.123*	
C10A	0.4535 (3)	−0.0307 (3)	−0.17793 (12)	0.0485 (7)	
H10A	0.3955	−0.1061	−0.1779	0.073*	
H10B	0.5462	−0.0483	−0.1576	0.073*	
H10C	0.4618	−0.0063	−0.2178	0.073*	
C11A	0.3980 (2)	−0.1770 (2)	−0.05835 (9)	0.0326 (6)	
C12A	0.3303 (2)	−0.2983 (3)	−0.04552 (14)	0.0577 (8)	
H12D	0.3754	−0.3321	−0.0089	0.087*	
H12E	0.3397	−0.3585	−0.0765	0.087*	
H12F	0.2314	−0.2838	−0.0428	0.087*	

### (1) (±)-N-[(1RS,2RS,4RS)-1,7,7-Trimethylbicyclo[2.2.1]heptan-2-yl]acetamide Atomic displacement parameters (Å^2^)

**Table:**

	*U*^11^	*U*^22^	*U*^33^	*U*^12^	*U*^13^	*U*^23^
O1	0.0203 (8)	0.0470 (12)	0.0600 (12)	−0.0052 (7)	0.0079 (7)	−0.0016 (9)
N1	0.0170 (9)	0.0367 (13)	0.0359 (12)	−0.0036 (9)	0.0059 (7)	−0.0039 (10)
C1	0.0598 (17)	0.0345 (17)	0.067 (2)	0.0022 (14)	0.0014 (13)	0.0058 (15)
C2	0.0429 (14)	0.0350 (16)	0.0646 (18)	0.0079 (12)	0.0082 (12)	−0.0045 (14)
C3	0.0343 (12)	0.0313 (15)	0.0397 (15)	−0.0006 (10)	0.0107 (10)	0.0004 (12)
C4	0.0247 (11)	0.0325 (15)	0.0356 (14)	−0.0007 (10)	0.0023 (9)	0.0018 (11)
C5	0.0426 (13)	0.0387 (16)	0.0396 (15)	−0.0083 (12)	0.0064 (10)	0.0103 (13)
C6	0.0446 (14)	0.0321 (16)	0.0540 (17)	−0.0119 (12)	0.0059 (11)	0.0012 (14)
C7	0.0433 (14)	0.0356 (16)	0.0362 (15)	−0.0025 (11)	0.0028 (10)	−0.0070 (13)
C8	0.0377 (13)	0.0527 (19)	0.0494 (17)	−0.0031 (12)	−0.0083 (11)	−0.0073 (14)
C9	0.0698 (18)	0.049 (2)	0.0551 (19)	−0.0046 (15)	0.0030 (14)	−0.0151 (16)
C10	0.0614 (16)	0.0407 (18)	0.0467 (17)	−0.0006 (13)	0.0242 (13)	0.0000 (14)
C11	0.0270 (12)	0.0370 (16)	0.0320 (14)	−0.0064 (11)	0.0064 (9)	−0.0031 (11)
C12	0.0338 (13)	0.0377 (17)	0.0558 (17)	−0.0037 (11)	0.0081 (11)	−0.0057 (13)
O1A	0.0206 (8)	0.0514 (12)	0.0522 (11)	0.0030 (7)	0.0057 (7)	0.0043 (9)
N1A	0.0180 (10)	0.0332 (13)	0.0384 (12)	−0.0016 (9)	0.0056 (7)	0.0044 (9)
C1A	0.0579 (17)	0.045 (2)	0.084 (2)	−0.0083 (14)	0.0174 (15)	−0.0010 (17)
C2A	0.0414 (14)	0.0380 (17)	0.078 (2)	−0.0099 (12)	0.0102 (13)	0.0029 (15)
C3A	0.0366 (13)	0.0381 (16)	0.0450 (16)	−0.0065 (11)	0.0084 (10)	−0.0007 (13)
C4A	0.0249 (11)	0.0332 (15)	0.0437 (15)	−0.0044 (10)	0.0001 (9)	−0.0020 (12)
C5A	0.0415 (14)	0.0390 (17)	0.0590 (18)	−0.0028 (12)	0.0089 (12)	−0.0067 (14)
C6A	0.0408 (15)	0.0461 (19)	0.085 (2)	0.0059 (13)	0.0128 (13)	0.0081 (18)
C7A	0.0384 (13)	0.0450 (17)	0.0410 (15)	−0.0032 (12)	0.0016 (10)	0.0135 (14)
C8A	0.0400 (14)	0.064 (2)	0.0507 (17)	−0.0095 (14)	−0.0097 (11)	0.0142 (16)
C9A	0.079 (2)	0.091 (3)	0.072 (2)	−0.009 (2)	−0.0030 (17)	0.040 (2)
C10A	0.0528 (15)	0.0471 (19)	0.0481 (16)	−0.0036 (13)	0.0164 (12)	0.0013 (14)
C11A	0.0262 (12)	0.0401 (16)	0.0324 (14)	−0.0002 (11)	0.0065 (9)	0.0024 (12)
C12A	0.0390 (14)	0.0480 (19)	0.086 (2)	0.0008 (13)	0.0093 (13)	0.0232 (17)

### (1) (±)-N-[(1RS,2RS,4RS)-1,7,7-Trimethylbicyclo[2.2.1]heptan-2-yl]acetamide Geometric parameters (Å, º)

**Table:**

O1—C11	1.237 (2)	O1A—C11A	1.236 (2)
N1—C11	1.338 (3)	N1A—C11A	1.334 (3)
N1—C4	1.451 (3)	N1A—C4A	1.452 (3)
N1—H1N	0.85 (2)	N1A—H1AN	0.87 (2)
C1—C6	1.536 (3)	C1A—C6A	1.529 (4)
C1—C2	1.547 (4)	C1A—C2A	1.529 (4)
C1—H1A	0.9900	C1A—H1A1	0.9900
C1—H1B	0.9900	C1A—H1A2	0.9900
C2—C3	1.539 (3)	C2A—C3A	1.552 (3)
C2—H2A	0.9900	C2A—H2A1	0.9900
C2—H2B	0.9900	C2A—H2A2	0.9900
C3—C10	1.508 (3)	C3A—C10A	1.494 (3)
C3—C4	1.550 (3)	C3A—C4A	1.536 (3)
C3—C7	1.556 (3)	C3A—C7A	1.560 (3)
C4—C5	1.557 (3)	C4A—C5A	1.588 (3)
C4—H4	1.0000	C4A—H4A	1.0000
C5—C6	1.528 (4)	C5A—C6A	1.523 (4)
C5—H5A	0.9900	C5A—H5A1	0.9900
C5—H5B	0.9900	C5A—H5A2	0.9900
C6—C7	1.541 (4)	C6A—C7A	1.542 (4)
C6—H6	1.0000	C6A—H6A	1.0000
C7—C8	1.534 (3)	C7A—C8A	1.530 (3)
C7—C9	1.537 (4)	C7A—C9A	1.543 (4)
C8—H8A	0.9800	C8A—H8A1	0.9800
C8—H8B	0.9800	C8A—H8A2	0.9800
C8—H8C	0.9800	C8A—H8A3	0.9800
C9—H9A	0.9800	C9A—H9A1	0.9800
C9—H9B	0.9800	C9A—H9A2	0.9800
C9—H9C	0.9800	C9A—H9A3	0.9800
C10—H10D	0.9800	C10A—H10A	0.9800
C10—H10E	0.9800	C10A—H10B	0.9800
C10—H10F	0.9800	C10A—H10C	0.9800
C11—C12	1.500 (3)	C11A—C12A	1.497 (3)
C12—H12A	0.9800	C12A—H12D	0.9800
C12—H12B	0.9800	C12A—H12E	0.9800
C12—H12C	0.9800	C12A—H12F	0.9800
			
C11—N1—C4	122.38 (18)	C11A—N1A—C4A	123.40 (18)
C11—N1—H1N	117.6 (16)	C11A—N1A—H1AN	119.6 (16)
C4—N1—H1N	119.6 (16)	C4A—N1A—H1AN	116.4 (16)
C6—C1—C2	102.5 (2)	C6A—C1A—C2A	102.8 (2)
C6—C1—H1A	111.3	C6A—C1A—H1A1	111.2
C2—C1—H1A	111.3	C2A—C1A—H1A1	111.2
C6—C1—H1B	111.3	C6A—C1A—H1A2	111.2
C2—C1—H1B	111.3	C2A—C1A—H1A2	111.2
H1A—C1—H1B	109.2	H1A1—C1A—H1A2	109.1
C3—C2—C1	104.02 (18)	C1A—C2A—C3A	103.87 (19)
C3—C2—H2A	111.0	C1A—C2A—H2A1	111.0
C1—C2—H2A	111.0	C3A—C2A—H2A1	111.0
C3—C2—H2B	111.0	C1A—C2A—H2A2	111.0
C1—C2—H2B	111.0	C3A—C2A—H2A2	111.0
H2A—C2—H2B	109.0	H2A1—C2A—H2A2	109.0
C10—C3—C2	114.18 (18)	C10A—C3A—C4A	114.5 (2)
C10—C3—C4	114.7 (2)	C10A—C3A—C2A	113.63 (19)
C2—C3—C4	104.27 (19)	C4A—C3A—C2A	104.2 (2)
C10—C3—C7	117.3 (2)	C10A—C3A—C7A	117.7 (2)
C2—C3—C7	100.96 (19)	C4A—C3A—C7A	103.72 (17)
C4—C3—C7	103.54 (16)	C2A—C3A—C7A	101.3 (2)
N1—C4—C3	116.15 (19)	N1A—C4A—C3A	117.1 (2)
N1—C4—C5	112.55 (17)	N1A—C4A—C5A	110.91 (17)
C3—C4—C5	103.13 (18)	C3A—C4A—C5A	103.01 (19)
N1—C4—H4	108.2	N1A—C4A—H4A	108.5
C3—C4—H4	108.2	C3A—C4A—H4A	108.5
C5—C4—H4	108.2	C5A—C4A—H4A	108.5
C6—C5—C4	102.74 (18)	C6A—C5A—C4A	101.5 (2)
C6—C5—H5A	111.2	C6A—C5A—H5A1	111.5
C4—C5—H5A	111.2	C4A—C5A—H5A1	111.5
C6—C5—H5B	111.2	C6A—C5A—H5A2	111.5
C4—C5—H5B	111.2	C4A—C5A—H5A2	111.5
H5A—C5—H5B	109.1	H5A1—C5A—H5A2	109.3
C5—C6—C1	107.8 (2)	C5A—C6A—C1A	107.5 (2)
C5—C6—C7	102.8 (2)	C5A—C6A—C7A	103.5 (2)
C1—C6—C7	102.75 (19)	C1A—C6A—C7A	103.8 (2)
C5—C6—H6	114.1	C5A—C6A—H6A	113.7
C1—C6—H6	114.1	C1A—C6A—H6A	113.7
C7—C6—H6	114.1	C7A—C6A—H6A	113.7
C8—C7—C9	106.4 (2)	C8A—C7A—C6A	115.7 (2)
C8—C7—C6	114.5 (2)	C8A—C7A—C9A	106.6 (2)
C9—C7—C6	113.5 (2)	C6A—C7A—C9A	113.4 (3)
C8—C7—C3	114.9 (2)	C8A—C7A—C3A	115.1 (2)
C9—C7—C3	114.28 (19)	C6A—C7A—C3A	92.51 (19)
C6—C7—C3	93.26 (18)	C9A—C7A—C3A	113.3 (2)
C7—C8—H8A	109.5	C7A—C8A—H8A1	109.5
C7—C8—H8B	109.5	C7A—C8A—H8A2	109.5
H8A—C8—H8B	109.5	H8A1—C8A—H8A2	109.5
C7—C8—H8C	109.5	C7A—C8A—H8A3	109.5
H8A—C8—H8C	109.5	H8A1—C8A—H8A3	109.5
H8B—C8—H8C	109.5	H8A2—C8A—H8A3	109.5
C7—C9—H9A	109.5	C7A—C9A—H9A1	109.5
C7—C9—H9B	109.5	C7A—C9A—H9A2	109.5
H9A—C9—H9B	109.5	H9A1—C9A—H9A2	109.5
C7—C9—H9C	109.5	C7A—C9A—H9A3	109.5
H9A—C9—H9C	109.5	H9A1—C9A—H9A3	109.5
H9B—C9—H9C	109.5	H9A2—C9A—H9A3	109.5
C3—C10—H10D	109.5	C3A—C10A—H10A	109.5
C3—C10—H10E	109.5	C3A—C10A—H10B	109.5
H10D—C10—H10E	109.5	H10A—C10A—H10B	109.5
C3—C10—H10F	109.5	C3A—C10A—H10C	109.5
H10D—C10—H10F	109.5	H10A—C10A—H10C	109.5
H10E—C10—H10F	109.5	H10B—C10A—H10C	109.5
O1—C11—N1	122.0 (2)	O1A—C11A—N1A	122.7 (2)
O1—C11—C12	121.4 (2)	O1A—C11A—C12A	121.1 (2)
N1—C11—C12	116.64 (18)	N1A—C11A—C12A	116.19 (19)
C11—C12—H12A	109.5	C11A—C12A—H12D	109.5
C11—C12—H12B	109.5	C11A—C12A—H12E	109.5
H12A—C12—H12B	109.5	H12D—C12A—H12E	109.5
C11—C12—H12C	109.5	C11A—C12A—H12F	109.5
H12A—C12—H12C	109.5	H12D—C12A—H12F	109.5
H12B—C12—H12C	109.5	H12E—C12A—H12F	109.5
			
C6—C1—C2—C3	−1.4 (3)	C6A—C1A—C2A—C3A	−1.5 (3)
C1—C2—C3—C10	163.3 (2)	C1A—C2A—C3A—C10A	163.7 (2)
C1—C2—C3—C4	−70.7 (2)	C1A—C2A—C3A—C4A	−71.1 (2)
C1—C2—C3—C7	36.5 (2)	C1A—C2A—C3A—C7A	36.4 (3)
C11—N1—C4—C3	91.9 (2)	C11A—N1A—C4A—C3A	91.2 (3)
C11—N1—C4—C5	−149.5 (2)	C11A—N1A—C4A—C5A	−151.0 (2)
C10—C3—C4—N1	−35.4 (3)	C10A—C3A—C4A—N1A	−38.7 (3)
C2—C3—C4—N1	−161.05 (18)	C2A—C3A—C4A—N1A	−163.36 (18)
C7—C3—C4—N1	93.7 (2)	C7A—C3A—C4A—N1A	91.0 (2)
C10—C3—C4—C5	−159.00 (19)	C10A—C3A—C4A—C5A	−160.7 (2)
C2—C3—C4—C5	75.4 (2)	C2A—C3A—C4A—C5A	74.6 (2)
C7—C3—C4—C5	−29.9 (2)	C7A—C3A—C4A—C5A	−31.1 (2)
N1—C4—C5—C6	−131.5 (2)	N1A—C4A—C5A—C6A	−131.0 (2)
C3—C4—C5—C6	−5.6 (2)	C3A—C4A—C5A—C6A	−4.9 (2)
C4—C5—C6—C1	−68.3 (2)	C4A—C5A—C6A—C1A	−69.5 (3)
C4—C5—C6—C7	39.7 (2)	C4A—C5A—C6A—C7A	39.9 (2)
C2—C1—C6—C5	73.3 (2)	C2A—C1A—C6A—C5A	74.5 (3)
C2—C1—C6—C7	−34.8 (3)	C2A—C1A—C6A—C7A	−34.7 (3)
C5—C6—C7—C8	63.2 (2)	C5A—C6A—C7A—C8A	62.4 (3)
C1—C6—C7—C8	175.1 (2)	C1A—C6A—C7A—C8A	174.6 (2)
C5—C6—C7—C9	−174.4 (2)	C5A—C6A—C7A—C9A	−173.9 (2)
C1—C6—C7—C9	−62.5 (3)	C1A—C6A—C7A—C9A	−61.7 (3)
C5—C6—C7—C3	−56.1 (2)	C5A—C6A—C7A—C3A	−57.1 (2)
C1—C6—C7—C3	55.8 (2)	C1A—C6A—C7A—C3A	55.1 (2)
C10—C3—C7—C8	60.6 (3)	C10A—C3A—C7A—C8A	60.7 (3)
C2—C3—C7—C8	−174.6 (2)	C4A—C3A—C7A—C8A	−66.9 (3)
C4—C3—C7—C8	−66.9 (2)	C2A—C3A—C7A—C8A	−174.8 (2)
C10—C3—C7—C9	−62.8 (3)	C10A—C3A—C7A—C6A	−179.3 (2)
C2—C3—C7—C9	62.0 (3)	C4A—C3A—C7A—C6A	53.1 (2)
C4—C3—C7—C9	169.7 (2)	C2A—C3A—C7A—C6A	−54.7 (2)
C10—C3—C7—C6	179.6 (2)	C10A—C3A—C7A—C9A	−62.4 (3)
C2—C3—C7—C6	−55.7 (2)	C4A—C3A—C7A—C9A	170.0 (2)
C4—C3—C7—C6	52.0 (2)	C2A—C3A—C7A—C9A	62.1 (3)
C4—N1—C11—O1	−1.1 (3)	C4A—N1A—C11A—O1A	−4.6 (3)
C4—N1—C11—C12	179.1 (2)	C4A—N1A—C11A—C12A	176.2 (2)

### (1) (±)-N-[(1RS,2RS,4RS)-1,7,7-Trimethylbicyclo[2.2.1]heptan-2-yl]acetamide Hydrogen-bond geometry (Å, º)

**Table:**

*D*—H···*A*	*D*—H	H···*A*	*D*···*A*	*D*—H···*A*
N1—H1*N*···O1*A*^i^	0.85 (2)	2.06 (2)	2.900 (2)	170 (2)
N1*A*—H1*AN*···O1^ii^	0.87 (2)	2.03 (2)	2.886 (2)	172 (2)
C8*A*—H8*A*1···O1^ii^	0.98	2.57	3.524 (3)	165
C12—H12*C*···O1^ii^	0.98	2.52	3.468 (3)	164

Symmetry codes: (i) −*x*+1, −*y*, −*z*; (ii) −*x*, −*y*, −*z*.

### (2) (±)-2-Chloro-N-[(1RS,2RS,4RS)-1,7,7-trimethylbicyclo[2.2.1]heptan-2-yl]acetamide Crystal data

**Table:**

C_12_H_20_ClNO	*F*(000) = 992
*M**_r_* = 229.74	*D*_x_ = 1.234 Mg m^−^^3^
Orthorhombic, *P**c**a**b*	Mo *K*α radiation, λ = 0.71073 Å
Hall symbol: -P 2bc 2ac	Cell parameters from 24915 reflections
*a* = 9.6852 (2) Å	θ = 1.0–30.0°
*b* = 10.7589 (3) Å	µ = 0.28 mm^−^^1^
*c* = 23.7261 (8) Å	*T* = 173 K
*V* = 2472.31 (12) Å^3^	Plate, colorless
*Z* = 8	0.35 × 0.10 × 0.09 mm

### (2) (±)-2-Chloro-N-[(1RS,2RS,4RS)-1,7,7-trimethylbicyclo[2.2.1]heptan-2-yl]acetamide Data collection

**Table:**

Nonius KappaCCD diffractometer	1854 reflections with *I* > 2σ(*I*)
Radiation source: fine-focus sealed tube	*R*_int_ = 0.097
Graphite monochromator	θ_max_ = 30.0°, θ_min_ = 2.6°
φ and ω scan	*h* = −13→13
6757 measured reflections	*k* = −15→15
3611 independent reflections	*l* = −33→33

### (2) (±)-2-Chloro-N-[(1RS,2RS,4RS)-1,7,7-trimethylbicyclo[2.2.1]heptan-2-yl]acetamide Refinement

**Table:**

Refinement on *F*^2^	Primary atom site location: structure-invariant direct methods
Least-squares matrix: full	Secondary atom site location: difference Fourier map
*R*[*F*^2^ > 2σ(*F*^2^)] = 0.073	Hydrogen site location: inferred from neighbouring sites
*wR*(*F*^2^) = 0.160	H atoms treated by a mixture of independent and constrained refinement
*S* = 1.02	*w* = 1/[σ^2^(*F*_o_^2^) + (0.0497*P*)^2^ + 1.7639*P*] where *P* = (*F*_o_^2^ + 2*F*_c_^2^)/3
3611 reflections	(Δ/σ)_max_ < 0.001
143 parameters	Δρ_max_ = 0.51 e Å^−^^3^
0 restraints	Δρ_min_ = −0.38 e Å^−^^3^

### (2) (±)-2-Chloro-N-[(1RS,2RS,4RS)-1,7,7-trimethylbicyclo[2.2.1]heptan-2-yl]acetamide Special details

**Table:**

Geometry. All e.s.d.'s (except the e.s.d. in the dihedral angle between two l.s. planes) are estimated using the full covariance matrix. The cell e.s.d.'s are taken into account individually in the estimation of e.s.d.'s in distances, angles and torsion angles; correlations between e.s.d.'s in cell parameters are only used when they are defined by crystal symmetry. An approximate (isotropic) treatment of cell e.s.d.'s is used for estimating e.s.d.'s involving l.s. planes.
Refinement. Refinement of *F*^2^ against ALL reflections. The weighted *R*-factor *wR* and goodness of fit *S* are based on *F*^2^, conventional *R*-factors *R* are based on *F*, with *F* set to zero for negative *F*^2^. The threshold expression of *F*^2^ > σ(*F*^2^) is used only for calculating *R*-factors(gt) *etc*. and is not relevant to the choice of reflections for refinement. *R*-factors based on *F*^2^ are statistically about twice as large as those based on *F*, and *R*- factors based on ALL data will be even larger.

### (2) (±)-2-Chloro-N-[(1RS,2RS,4RS)-1,7,7-trimethylbicyclo[2.2.1]heptan-2-yl]acetamide Fractional atomic coordinates and isotropic or equivalent isotropic displacement parameters (Å^2^)

**Table:**

	*x*	*y*	*z*	*U*_iso_*/*U*_eq_	
Cl1	0.83924 (7)	1.01572 (7)	0.43765 (4)	0.0460 (3)	
O1	0.68852 (15)	0.76668 (17)	0.44335 (8)	0.0326 (5)	
N1	0.8914 (2)	0.6671 (2)	0.42922 (10)	0.0241 (5)	
H1	0.972 (3)	0.673 (2)	0.4329 (11)	0.026 (8)*	
C1	0.7790 (3)	0.3051 (3)	0.36991 (15)	0.0436 (8)	
H1A	0.7757	0.2399	0.3417	0.052*	
H1B	0.7467	0.2723	0.4056	0.052*	
C2	0.6933 (3)	0.4183 (3)	0.35161 (14)	0.0377 (7)	
H2A	0.6194	0.4345	0.3782	0.045*	
H2B	0.6539	0.4055	0.3145	0.045*	
C3	0.7980 (2)	0.5264 (2)	0.35096 (12)	0.0292 (6)	
C4	0.8313 (2)	0.5475 (2)	0.41407 (11)	0.0260 (6)	
H4	0.7456	0.5373	0.4355	0.031*	
C5	0.9296 (3)	0.4360 (2)	0.42903 (13)	0.0332 (7)	
H5A	1.0224	0.4647	0.4373	0.040*	
H5B	0.8950	0.3891	0.4609	0.040*	
C6	0.9261 (3)	0.3591 (3)	0.37543 (15)	0.0409 (8)	
H6	0.9999	0.2970	0.3727	0.049*	
C7	0.9285 (3)	0.4580 (3)	0.32796 (12)	0.0321 (7)	
C8	1.0598 (3)	0.5389 (3)	0.32675 (14)	0.0435 (8)	
H8A	1.1362	0.4900	0.3136	0.065*	
H8B	1.0792	0.5688	0.3640	0.065*	
H8C	1.0458	0.6082	0.3019	0.065*	
C9	0.9126 (4)	0.4040 (4)	0.26906 (16)	0.0626 (11)	
H9A	0.9126	0.4701	0.2419	0.094*	
H9B	0.8271	0.3592	0.2667	0.094*	
H9C	0.9880	0.3486	0.2614	0.094*	
C10	0.7496 (3)	0.6398 (3)	0.31989 (14)	0.0427 (8)	
H10A	0.7357	0.6197	0.2809	0.064*	
H10B	0.8179	0.7042	0.3230	0.064*	
H10C	0.6642	0.6682	0.3359	0.064*	
C11	0.8159 (2)	0.7647 (2)	0.44464 (11)	0.0239 (6)	
C12	0.8982 (2)	0.8735 (2)	0.46654 (14)	0.0324 (7)	
H12A	0.9947	0.8620	0.4571	0.039*	
H12B	0.8906	0.8767	0.5073	0.039*	

### (2) (±)-2-Chloro-N-[(1RS,2RS,4RS)-1,7,7-trimethylbicyclo[2.2.1]heptan-2-yl]acetamide Atomic displacement parameters (Å^2^)

**Table:**

	*U*^11^	*U*^22^	*U*^33^	*U*^12^	*U*^13^	*U*^23^
Cl1	0.0364 (4)	0.0305 (4)	0.0711 (6)	−0.0062 (3)	−0.0118 (4)	0.0006 (4)
O1	0.0136 (8)	0.0345 (10)	0.0499 (13)	0.0001 (7)	−0.0002 (8)	−0.0073 (10)
N1	0.0120 (10)	0.0280 (11)	0.0323 (14)	−0.0021 (8)	−0.0006 (9)	−0.0028 (10)
C1	0.0349 (15)	0.0323 (15)	0.063 (2)	−0.0059 (12)	−0.0009 (15)	−0.0040 (16)
C2	0.0213 (13)	0.0361 (16)	0.056 (2)	−0.0064 (11)	0.0011 (12)	−0.0065 (15)
C3	0.0238 (12)	0.0276 (14)	0.0363 (17)	−0.0023 (10)	−0.0014 (11)	−0.0005 (13)
C4	0.0203 (12)	0.0248 (13)	0.0329 (16)	−0.0026 (10)	0.0057 (11)	0.0000 (11)
C5	0.0273 (13)	0.0285 (13)	0.0439 (18)	0.0001 (11)	0.0000 (12)	0.0083 (14)
C6	0.0261 (14)	0.0277 (15)	0.069 (2)	0.0058 (11)	−0.0011 (14)	−0.0065 (15)
C7	0.0247 (13)	0.0378 (16)	0.0338 (17)	−0.0059 (11)	0.0055 (11)	−0.0111 (14)
C8	0.0324 (15)	0.054 (2)	0.0442 (19)	−0.0137 (14)	0.0148 (14)	−0.0130 (16)
C9	0.0484 (19)	0.078 (3)	0.062 (3)	−0.0176 (18)	0.0151 (17)	−0.035 (2)
C10	0.0449 (17)	0.0419 (17)	0.0412 (19)	0.0003 (15)	−0.0156 (14)	−0.0008 (15)
C11	0.0161 (11)	0.0268 (13)	0.0289 (15)	−0.0006 (9)	−0.0008 (10)	−0.0028 (12)
C12	0.0180 (12)	0.0304 (14)	0.0488 (19)	0.0023 (10)	−0.0066 (12)	−0.0108 (14)

### (2) (±)-2-Chloro-N-[(1RS,2RS,4RS)-1,7,7-trimethylbicyclo[2.2.1]heptan-2-yl]acetamide Geometric parameters (Å, º)

**Table:**

Cl1—C12	1.771 (3)	C5—H5A	0.9700
O1—C11	1.235 (3)	C5—H5B	0.9700
N1—C11	1.331 (3)	C6—C7	1.550 (4)
N1—C4	1.457 (3)	C6—H6	0.9800
N1—H1	0.78 (3)	C7—C9	1.522 (4)
C1—C2	1.536 (4)	C7—C8	1.541 (4)
C1—C6	1.544 (4)	C8—H8A	0.9600
C1—H1A	0.9700	C8—H8B	0.9600
C1—H1B	0.9700	C8—H8C	0.9600
C2—C3	1.543 (4)	C9—H9A	0.9600
C2—H2A	0.9700	C9—H9B	0.9600
C2—H2B	0.9700	C9—H9C	0.9600
C3—C10	1.501 (4)	C10—H10A	0.9600
C3—C4	1.548 (4)	C10—H10B	0.9600
C3—C7	1.561 (4)	C10—H10C	0.9600
C4—C5	1.573 (4)	C11—C12	1.508 (3)
C4—H4	0.9800	C12—H12A	0.9700
C5—C6	1.517 (4)	C12—H12B	0.9700
			
C11—N1—C4	123.0 (2)	C5—C6—H6	114.2
C11—N1—H1	117 (2)	C1—C6—H6	114.2
C4—N1—H1	119 (2)	C7—C6—H6	114.2
C2—C1—C6	102.9 (2)	C9—C7—C8	106.4 (2)
C2—C1—H1A	111.2	C9—C7—C6	113.8 (3)
C6—C1—H1A	111.2	C8—C7—C6	114.4 (2)
C2—C1—H1B	111.2	C9—C7—C3	114.8 (2)
C6—C1—H1B	111.2	C8—C7—C3	114.1 (2)
H1A—C1—H1B	109.1	C6—C7—C3	93.3 (2)
C1—C2—C3	104.2 (2)	C7—C8—H8A	109.5
C1—C2—H2A	110.9	C7—C8—H8B	109.5
C3—C2—H2A	110.9	H8A—C8—H8B	109.5
C1—C2—H2B	110.9	C7—C8—H8C	109.5
C3—C2—H2B	110.9	H8A—C8—H8C	109.5
H2A—C2—H2B	108.9	H8B—C8—H8C	109.5
C10—C3—C2	114.3 (2)	C7—C9—H9A	109.5
C10—C3—C4	114.9 (2)	C7—C9—H9B	109.5
C2—C3—C4	103.7 (2)	H9A—C9—H9B	109.5
C10—C3—C7	117.7 (2)	C7—C9—H9C	109.5
C2—C3—C7	100.4 (2)	H9A—C9—H9C	109.5
C4—C3—C7	103.8 (2)	H9B—C9—H9C	109.5
N1—C4—C3	116.8 (2)	C3—C10—H10A	109.5
N1—C4—C5	112.1 (2)	C3—C10—H10B	109.5
C3—C4—C5	103.4 (2)	H10A—C10—H10B	109.5
N1—C4—H4	108.0	C3—C10—H10C	109.5
C3—C4—H4	108.0	H10A—C10—H10C	109.5
C5—C4—H4	108.0	H10B—C10—H10C	109.5
C6—C5—C4	102.3 (2)	O1—C11—N1	123.8 (2)
C6—C5—H5A	111.3	O1—C11—C12	121.5 (2)
C4—C5—H5A	111.3	N1—C11—C12	114.63 (19)
C6—C5—H5B	111.3	C11—C12—Cl1	111.55 (18)
C4—C5—H5B	111.3	C11—C12—H12A	109.3
H5A—C5—H5B	109.2	Cl1—C12—H12A	109.3
C5—C6—C1	107.3 (2)	C11—C12—H12B	109.3
C5—C6—C7	103.6 (2)	Cl1—C12—H12B	109.3
C1—C6—C7	102.1 (2)	H12A—C12—H12B	108.0
			
C6—C1—C2—C3	−2.3 (3)	C1—C6—C7—C9	−63.4 (3)
C1—C2—C3—C10	164.5 (3)	C5—C6—C7—C8	62.6 (3)
C1—C2—C3—C4	−69.6 (3)	C1—C6—C7—C8	174.0 (2)
C1—C2—C3—C7	37.5 (3)	C5—C6—C7—C3	−55.8 (2)
C11—N1—C4—C3	91.6 (3)	C1—C6—C7—C3	55.6 (2)
C11—N1—C4—C5	−149.3 (2)	C10—C3—C7—C9	−62.8 (3)
C10—C3—C4—N1	−35.2 (3)	C2—C3—C7—C9	61.9 (3)
C2—C3—C4—N1	−160.7 (2)	C4—C3—C7—C9	168.9 (2)
C7—C3—C4—N1	94.7 (2)	C10—C3—C7—C8	60.3 (3)
C10—C3—C4—C5	−158.8 (2)	C2—C3—C7—C8	−175.0 (3)
C2—C3—C4—C5	75.6 (2)	C4—C3—C7—C8	−67.9 (3)
C7—C3—C4—C5	−28.9 (2)	C10—C3—C7—C6	179.0 (2)
N1—C4—C5—C6	−132.9 (2)	C2—C3—C7—C6	−56.3 (2)
C3—C4—C5—C6	−6.2 (2)	C4—C3—C7—C6	50.8 (2)
C4—C5—C6—C1	−67.7 (3)	C4—N1—C11—O1	−6.0 (4)
C4—C5—C6—C7	39.8 (2)	C4—N1—C11—C12	171.7 (2)
C2—C1—C6—C5	74.5 (3)	O1—C11—C12—Cl1	−47.2 (3)
C2—C1—C6—C7	−34.1 (3)	N1—C11—C12—Cl1	135.1 (2)
C5—C6—C7—C9	−174.8 (2)		

### (2) (±)-2-Chloro-N-[(1RS,2RS,4RS)-1,7,7-trimethylbicyclo[2.2.1]heptan-2-yl]acetamide Hydrogen-bond geometry (Å, º)

**Table:**

*D*—H···*A*	*D*—H	H···*A*	*D*···*A*	*D*—H···*A*
N1—H1···O1^i^	0.79 (3)	2.21 (3)	2.983 (2)	168 (2)
C12—H12*A*···O1^i^	0.97	2.36	3.238 (3)	151

Symmetry code: (i) *x*+1/2, −*y*+3/2, *z*.
